# The Effect of the Hydroalcoholic Extract of Watercress on the Levels of Protein Carbonyl, Inflammatory Markers, and Vitamin E in Chronic Hemodialysis Patients

**DOI:** 10.1155/2021/5588464

**Published:** 2021-05-26

**Authors:** Moslem Sedaghattalab, Marzieh Razazan, Mohsen Shahpari, Nahid Azarmehr, Rozina Abbasi Larki, Hossein Sadeghi, Arash Asfaram, Tahere Taheri, Aminollah Pourshohod, Zahra Moslemi, Kazem Abbaszadeh-Goudarzi, Amir Hossein Doustimotlagh

**Affiliations:** ^1^Department of Internal Medicine, Yasuj University of Medical Sciences, Yasuj, Iran; ^2^Student Research Committee, Yasuj University of Medical Sciences, Yasuj, Iran; ^3^Department of Nephrology, Yasuj University of Medical Sciences, Yasuj, Iran; ^4^Medicinal Plants Research Center, Yasuj University of Medical Sciences, Yasuj, Iran; ^5^Medical-Surgical Nursing Department, Nursing Faculty, Yasuj University of Medical Science, Yasuj, Iran; ^6^Cellular and Molecular Research Center, Sabzevar University of Medical Sciences, Sabzevar, Iran; ^7^Department of Clinical Biochemistry, Faculty of Medicine, Yasuj University of Medical Sciences, Yasuj, Iran

## Abstract

**Introduction:**

Chronic kidney disorder is a main public health concern. Inflammatory processes and oxidative stress are common in end-stage renal disease patients. We aimed to evaluate the effect of the hydroalcoholic extract of watercress (WC) on the inflammatory cytokines and protein carbonyl (PCO) contents in chronic hemodialysis patients.

**Methods:**

This was a double-blind randomized clinical trial performed on 46 hemodialysis patients. The participants were randomly divided into two groups: intervention group (500 mg hydroalcoholic extract of WC every day for 4 weeks) and control group (500 mg of white flour every night for 4 weeks). The blood samples were taken to determine the levels of vitamin E, PCO, and inflammatory cytokines at baseline and the end of treatment.

**Results:**

Forty-five patients completed the study (22 patients in the intervention group and 23 patients in the control group). There was a significant reduction in the PCO level (20.33 ± 4.40 vs. 15.06 ± 6.41, *P*=0.001) in the intervention group; also, this change was statistically significant relative to the control group. Furthermore, there were significant reductions in hs-CRP (8953.30 ± 5588.06 vs. 7249.86 ± 5091.62, *P*=0.007) and IL-6 (60.10 (55.99, 73.10) vs. 55.21 (53.39, 60.48), *P*=0.050) in the intervention group, but these changes were not significant in comparison with the control group.

**Conclusion:**

We conclude that the hydroalcoholic extract of WC reduced the PCO content in hemodialysis patients via inhibition of protein oxidation. Although WC administration had caused a significant reduction in IL-6 and CRP levels, these differences were not statistically significant relative to the control group. Further research is needed to identify the antioxidant and anti-inflammatory effects of WC in hemodialysis patients.

## 1. Introduction

Chronic kidney disease (CKD) is a main public health concern and an important factor in reducing life expectancy [1]. With the quick rise in the incidence of end-stage renal disease (ESRD), the number of renal replacement therapies (RRTs) is steadily increasing. RRT, which includes dialysis and kidney transplantation, is a treatment to extend the lifespan of ESRD patients [2]. Hemodialysis is the major therapy in the controlling of CKD, which can destroy vitamins, water-soluble peptides, and amino acids [3]. Dialysis is required when the glomerular filtration rate (GFR) in ESRD patients is reduced to 10 to 15 mL/min per 1.73 m^2^ [4, 5].

Increased oxidative stress in dialysis patients is due to the loss of antioxidant compounds during dialysis, bacterial products during dialysis, and malnutrition in these patients [6]. Over the last two decades, oxidative stress has been a risk factor for mortality in CKD patients [7]. In a normal cell, there is a good balance between oxidants such as reactive oxygen species (ROS) and antioxidants; increasing pro-oxidants or decreasing antioxidants led to oxidative stress that it able to cause serious cellular damage [8]. In the hemodialysis patients, oxidative stress increases [9]; then, oxidative compounds can interact with cellular components such as lipids, proteins, and DNA by various reactions and subsequently causes pathological complications [10].

Dialysis patients are known to have a chronic inflammatory condition [11], and inflammatory processes are common in CKD and ESRD patients [12]. Although inflammation is a frequent feature of dialysis patients, its underlying factors and mechanisms are poorly understood [13]. Inflammation in these patients may occur due to dialysis-related factors (such as exposure to endotoxins and cytokine inducers in dialysate) and nondialysis-related factors (such as genetic factors, infection, and other underlying diseases) [14].

Increased oxidative stress and inflammation in hemodialysis patients lead to cardiovascular disease (CVD) [15]; in other words, inflammation as a risk factor for CVD is a main cause of mortality in hemodialysis patients [16]. Studies have shown that proinflammatory cytokines such as IL-6, IL-1, and TNF-*α* increase in dialysis patients [16, 17]. IL-6 is a proinflammatory cytokine that induces hepatic C-reactive protein (CRP) production and eventually leads to CVD [13, 17].

Watercress (*Nasturtium officinale*, WC) is a plant that grows in arid and aquatic situations and is rich in vitamins (such as A, B, C, K, E, and folic acid), ions/elements (such as iron, chromium, calcium, magnesium, phosphorus, iron, potassium, zinc, and sodium), and bioactive substances (for instance, *β*-carotene, lutein, and quercetin). It is used to treat diabetes, anemia, and eczema, as well as renal and hepatic disorders [18–20]. Previous studies showed that WC reduces the risk of colon, lung, lymphatic, and prostate cancers [21, 22]. In addition, in vivo and in vitro studies have reported that WC has antidiabetic [23, 24], anti-inflammatory [25], antioxidant [26–28], nephroprotective [29], and hepatoprotective effects [30, 31].

Therefore, based on anti-inflammatory and antioxidant properties of WC, the purpose of the present study was to evaluate the effect of the hydroalcoholic extract of WC on the inflammatory cytokines and protein carbonyl contents in chronic hemodialysis patients.

## 2. Materials and Methods

### 2.1. Chemicals

Trichloroacetic acid (TCA), guanidinium chloride (GdnHCl), 2,4-dinitrophenylhydrazine (DNPH), acetic acid, acetonitrile, acetate sodium, and methanol were obtained from Merck (Germany). All other reagents and chemicals utilized were of analytical grade.

### 2.2. Plant Material and Extraction

Aerial parts of WC were provided in March–May 2019 from the Shehniz area placed in Yasuj, Iran. The WC plant was identified by the botanist (Herbarium No. HYU30230). The aerial parts of the plant were dried in the shade at 25°C and finely ground. Briefly, 100 g powder of the plant was suspended in 70% ethanol (1000 ml) at 25°C for 48 hours. The extract was filtered by a filter paper, transferred to a vacuum distillation apparatus, and concentrated as far as possible. Then, the extract was dried in a 50°C incubator and stored at −20°C.

### 2.3. Clinical Trial

This double-blind randomized clinical trial was performed on chronic hemodialysis patients in Shahid Beheshti Hospital, Yasuj University of Medical Sciences, between July 2019 and August 2019. The study was permitted by the Research Committee of Yasuj University of Medical Sciences (Ethical code: YUMS.REC.1398.112); then, it was confirmed in the Iranian Clinical Trial System (http://www.irct.ir) with the registration number IRCT20201228049866N1. Before starting the study, all patients completed an informed consent form. Ethical considerations include confidentiality of information, imposing no cost on subjects and assigning code to each subject. Inclusion criteria were ≥18 years, ≥3 months on hemodialysis and three times a week hemodialysis, not taking medications like corticosteroids and nonsteroidal anti-inflammatory drugs (NSAIDs) at least for 4 weeks, and no acute infectious or inflammatory disease. Exclusion criteria were as follows: (1) active hepatic disease, malignancy, AIDS, rheumatic diseases, and other inflammatory diseases; (2) presence of any infection in late two months; (3) rejected renal transplantation; (4) allergy to the hydroalcoholic extract of WC; (5) having active infectious disease during the study; (6) pre-existing cardiac arrhythmias; (7) having of hypotension; (8) immunodeficiency; (9) malnutrition and cachexia (BMI <18.5 kg/m^2^); (10) albumin ≤3 mg/dL; (11) patient's displeasure to continue the study; and (12) kidney transplantation.

From the 110 hemodialysis patients, 46 patients were selected using simple random sampling. The mean year that the patients have been in the hemodialysis unit was about 2 years. The patients were randomly (with 1 : 1 ratio) divided into two groups: intervention (*n* = 23) and control (*n* = 23). During a four weeks' period, the patients in the intervention group took 500 mg hydroalcoholic extract of WC once a day and those in the control group received drug-like (capsule color, packing) containing 500 mg of white flour. Hydroalcoholic extract of WC dose was selected according to previous clinical and pharmacologic studies [3, 21]. To confirm that the supplement was taken, the patient was asked to bring the capsule bottle to the next dialysis session. Patients were recommended not to consume any other nutritional or antioxidant supplements during the trial in order to sustain their daily dietary patterns, diet, and physical activity. The supplementation and placebo were packaged in similar color, form, wrapping, and size. The patients were checked for WC side effects based on the dialysis frequency of at least three times a week. Patients were monitored weekly for drug use for the assurance of clinical research, and they were asked to bring the empty container.

A 5 mL blood sample was taken (when the dialysis catheter was attached to the patient) at baseline and the end of treatment for measurement of NO (nitric oxide) metabolite, protein carbonyl (PCO), vitamin E, and inflammatory markers (such as TNF-*α*, IL-6, and hs-CRP).

### 2.4. HPLC Conditions

Vitamin E in a standard and hydroalcoholic extract of WC was analyzed quantitatively by reverse-phase HPLC via a Knauer column (4.6 mm diameter and 250 mm length) with a precolumn (particle size of 5 *μ*m, Eurospher 100-5 C18) at 220 nm. The final concentrations of all experiments were recorded based on the mobile phase at room temperature and were a mixture of acetonitrile (95%) and water (5.0%) at a flow rate of 1.0 mL·min^−1^, and the injection loop (20 *μ*L). For the quantification of vitamin E, blood samples of chronic hemodialysis patients were centrifuged at 3000 rpm for 15 min and the serum was then separated. The proteins were precipitated by adding 200 *μ*L of acetonitrile to 100 *µ*L serum in 1 mL microtubes. After centrifugation (15 min, 10000 rpm), 20 *μ*L of the supernatant was injected into the HPLC-UV system for subsequent analysis. The concentration of vitamin E in the samples was determined from the calibration curve.

### 2.5. Determination of Nitric Oxide Metabolite

The nitrite level was measured as an index of NO production based on the Griess reaction [32]. The NO metabolite level was presented as *µ*mol/L utilizing sodium nitrite as standard (0–100 *μ*mol/L).

### 2.6. Determination of the Protein Carbonyl Level

The carbonyl content of the protein was calculated by a spectrophotometric method [33]. After treatment with DNPH (10 mmol/L) in HCl (2 mol/L) and 50% TCA, the deposit was washed with a mixture of ethanol and ethyl acetate (1 : 1, *v*/*v*) and dissolved in GdnHCl (6 mol/L). At the end of the procedure, the PCO level was determined using the molar absorption coefficient of 2.2 × 10^4^ M^−1^·cm^−1^ and presented as *μ*mol/mg protein.

### 2.7. Determination of Inflammatory Markers

In serum samples on 0^th^ and 28^th^ days, the levels of hs-CRP, IL-6, and TNF-*α* were measured using ELISA kits (Karmania Pars Gene, Kerman, Iran) based on the manufacturer's guidelines. The lower detection limit was 2 pg/mL for TNF-*α*, 3 pg/mL for IL-6, and 10 ng/mL for hs-CRP. The intra- and inter assay CVs were 10 and 12% for hs-CRP, 3 and 9% for IL-6, and 3 and 8% for TNF-*α*, respectively. Absorption was measured using an ELISA reader (BioTek, Winooski, Vermont, USA) at 450 nm.

### 2.8. Statistical Analyses

Results are presented as mean ± SD or median (25th to 75th interquartile range). The normality test was performed to select the appropriate statistical test. Parametric tests were used for normal distribution, and nonparametric tests were used for data without normal distribution. Baseline data were evaluated to determine the possible significant intergroup variations. An independent *t*-test and paired *t*-test were used for parametric data; also, the Mann–Whitney *U* test and Wilcoxon signed-rank test were used for nonparametric data. The significance level was assumed as *P* < 0.05.

## 3. Results

**Figure 1:**
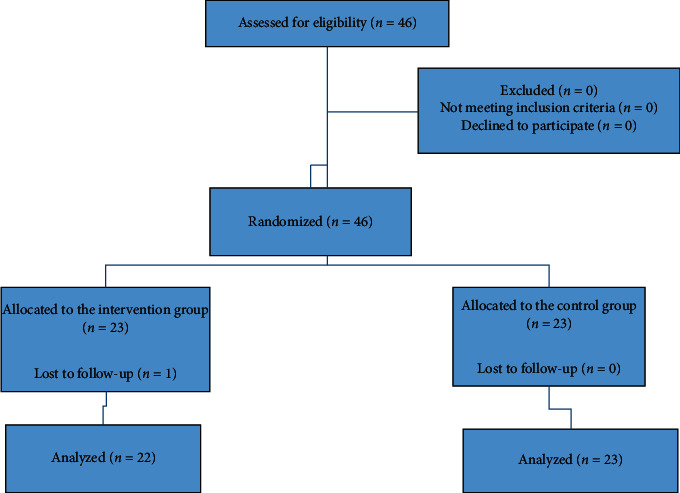
Schematic flow diagram of the study.

The flow diagram of the present study is shown in Figure 1. Out of 46 hemodialysis patients, 23 patients entered in each group (23 patients in the intervention group and 23 patients in the control group). Among the patients of the intervention group, one patient left the study due to kidney transplantation. Finally, 22 patients in the intervention group (12 males and 10 females) and 23 patients in the control group (13 males and 10 females) completed the study. In the current study, the main cause of renal failure was diabetes in 22 (47.82%) patients. One patient showed some gastrointestinal symptoms of pain and diarrhea that were tolerable, and the patient continued the study.

### 3.1. RP-HPLC Results

**Figure 2:**
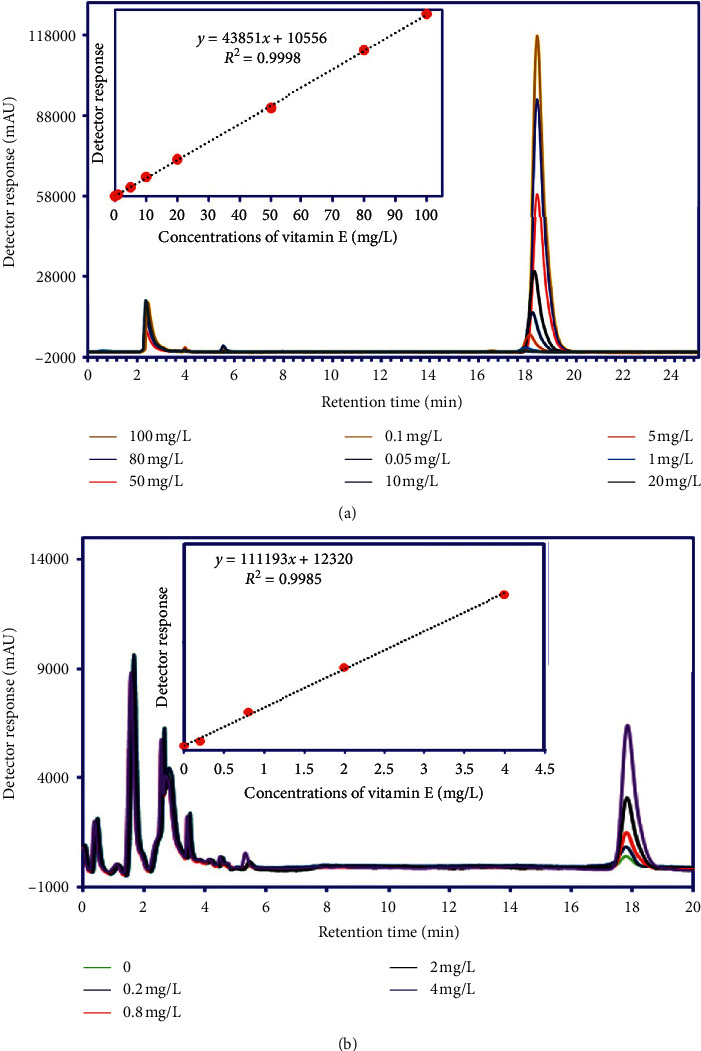
The typical chromatograms and calibration curves obtained from standard vitamin E (a) and vitamin E in the hydroalcoholic extract of WC by the standard addition method (b).

The RP-HPLC quantitative analyses were carried out by the standard addition method. The typical chromatograms and calibration curve of vitamin E and the hydroalcoholic extract of WC are displayed in Figures 2(a) and 2(b), respectively. The retention time (*t*_*R*_) of vitamin E was 18.1 ± 0.20 min. The linear range of injected concentrations in the standard and hydroalcoholic extract of WC was obtained in the range of 0.0–100 mg·L^−1^ (correlation coefficient 0.999). Finally, on the basis of obtained data on injected samples of the hydroalcoholic extract of WC, the amount of vitamin E was measured as 52.0 ± 2.0 mg·g^−1^.

### 3.2. Baseline Characteristics

Baseline data of the patients are presented in Table 1. The baseline data showed that there was no statistically significant change among the variables between intervention and control groups.

### 3.3. Effects of the WC Extract on NO Metabolite and PCO Levels

Table 2 shows the comparisons of NO metabolite and PCO variables between groups at the beginning and the end of the study. There was no statistically significant change between groups regarding NO metabolite. There was a significant reduction in the PCO level (20.33 ± 4.40 vs. 15.06 ± 6.41, *P*=0.001) in the intervention group; also, this change was statistically significant relative to the control group.

### 3.4. Effects of the WC Extract on Inflammatory Markers

Differences between intervention and control groups in inflammatory markers at the end of the study are indicated in Table 2. There was a significant reduction in hs-CRP (8953.30 ± 5588.06 vs. 7249.86 ± 5091.62, *P*=0.007) and IL-6 (60.10 (55.99, 73.10) vs. 55.21 (53.39, 60.48), *P*=0.050) in the intervention group, but these change was not significant in comparison with the control group. No statistically significant change was observed between intervention and control groups in hs-CRP (*P*=0.785), IL-6 (*P*=0.196), and TNF-*α* (*P*=0.276) levels.

### 3.5. Effects of the WC Extract on the Vit E Level

Table 2 shows the Vit E level of the participants at the baseline and after 28 days of taking the WC extract or without taking the WC extract (control). No significant change in the Vit E level was determined between WC extracts and control groups.

## 4. Discussion

Oxidative stress is defined as an imbalance between oxidant and antioxidant molecules. Oxidant products include ROS (such as superoxide and hydroxyl radical) and reactive nitrogen species (such as nitric oxide (NO)), while antioxidant molecules include Vit C and E, glutathione, and antioxidant enzymes [9]. Oxidative stress in CKD patients is mediated by several factors such as chronic inflammation, low-molecular-weight uremic toxins, elevated homocysteine level, nutritional inadequacy, and anemia [34, 35].

Oxidative stress is occurring during renal failure and hemodialysis, and it may lead to protein oxidation. The formation of PCO in proteins happens by direct oxidation via ROS, with the final production of oxidized amino acids [35]. To the best of our knowledge, the current study is the first work carried out to investigate the effects of the WC extract on serum levels of PCO and inflammatory markers in hemodialysis patients.

Agreeing with the current results, some studies have indicated that the PCO content increased significantly in hemodialysis and chronic renal failure groups relative to the controls [35–39]. Our finding indicated that the serum PCO contents (a well-confirmed marker of protein oxidation) significantly increased in the control group during the study; this result showed that there is a low-molecular-weight dialyzable substance in the serum of patients on chronic hemodialysis [39] that may lead to protein oxidation.

The main finding of the current study is that the consumption of WC extracts (a dose of 500 mg) for 28 days significantly decreased PCO levels in hemodialysis patients in the intervention group. Furthermore, daily consumption of WC extracts was associated with a marked reduction in serum levels of PCO in the intervention group compared with control subjects. The use of the WC extract in gentamicin- (GM-) induced and vancomycin- (VCM-) induced nephrotoxicity has been studied in animal models [29, 40]. Shahani et al. concluded that treatment with the WC extract (100 and 200 mg/kg) significantly reduced the ROS formation and serum levels of blood urea nitrogen and creatinine and modulated the pathological changes in kidney tissue [40]. Karami et al. revealed that administration of the WC extract (500 mg/kg) significantly decreased the levels of uric acid, creatinine, and malondialdehyde in the blood and kidney in VCM-induced nephrotoxicity [29]. Furthermore, Bahramikia et al. showed that adding Fe^2+^/ascorbic acid to the liver homogenate markedly augmented ROS and PCO production, while the WC extract (0.1 mL) showed preventing activity against ROS and PCO formation [41]. Also, a previous study demonstrated that the WC extract significantly diminished the PCO level in the liver tissue of bile duct-ligated rats [42]. The strong antioxidant activity of WC has been attributed to various mechanisms such as direct trapping of ROS, removal of peroxides, inhibition of lipid peroxidation, binding to transition metal ion, and inhibition of chain beginning and reductive capacity [43–45].

The use of WC extracts as an anti-inflammatory factor has been studied in various animal models [25, 40, 46]. Shahani et al. indicated that the WC extract (100 and 200 mg/kg) markedly reduced the levels of TNF-*α* and NO in GM-induced nephrotoxicity [40]. In another study, the WC extract and WC gel decreased the inflammatory cells infiltration and proinflammatory cytokines (such as macrophage inflammatory protein 2 and IL-1*β*) levels in acute inflammation induced by croton oil [46]. The current study showed significant decreases in serum CRP and IL-6 levels in the intervention group after 4 weeks. Of note, these changes were not statistically significant between intervention and control groups. The insignificant change of inflammation markers between intervention and control groups in the present study may be the result of small sample size, duration of WC extract consumption, and variation in the WC extract type.

Forgarty et al. observed that the lipid-soluble antioxidants such as *α*-tocopherol, *γ*-tocopherol, and xanthophyll increased after WC consumption [47]. Gill et al. showed that the consumption of 85 g of raw WC for 8 weeks significantly increased serum antioxidants (*β*-carotene and lutein) compared with the control group [21]. Also, they indicated that WC can increase lipid and aqueous soluble antioxidants in healthy participants as follows: *α*-tocopherol (26%), *β*-carotene (33%), and ascorbic acid (35%) [21]. However, our findings showed no significant change in the Vit E level between WC extract and control groups.

## 5. Conclusion

In summary, our results indicated that the hydroalcoholic extract of WC reduced the PCO content in hemodialysis patients via inhibition of protein oxidation. Although WC administration had caused significant reductions in IL-6 and CRP levels, these differences were not statistically significant relative to the control group. Further research is needed to identify the antioxidant and anti-inflammatory effects of WC in hemodialysis patients and to improve therapeutic approaches to decrease oxidative stress.

## Figures and Tables

**Table 1:** Baseline characteristics of control and intervention groups.

Variable	Control	Intervention	*P* value
Age	63.08 ± 13.9	58.86 ± 16.68	0.357
BMI (kg/m^2^)	23.79 ± 4.17	25.59 ± 4.25	0.153
hs-CRP	10508.52 ± 5479.98	8953.30 ± 5588.06	0.346
PCO	22.19 ± 4.26	20.33 ± 4.40	0.153
NO metabolite	9.12 ± 5.88	12.25 ± 7.66	0.142
TNF-*α* (pg/mL)	15.95 (15.17, 16.66)	15.96 (15.16, 16.66)	0.785
IL-6 (pg/mL)	58.24 (55.19, 61.12)	60.10 (55.99, 73.10)	0.075
Vit E	740.00 ± 104.12	703.05 ± 73.91	0.186

Values are mean ± SD for the data with normal distribution and median (interquartile ranges) for the data not normally distributed. BMI: body mass index; CRP: C-reactive protein; IL-6: interleukin-6; TNF-*α*: tumor necrosis factor-*α*; PCO: protein carbonyl; NO metabolite: nitric oxide metabolite; Vit E: vitamin E.

**Table 2:** Comparison of changes in the oxidant-antioxidant parameters and inflammatory markers during the study period between the intervention and control groups.

Variable		Baseline	After 4 weeks	*P* value	Net differences of groups	*P* value
PCO	Control	22.19 ± 4.26	26.46 ± 7.72	0.022	4.26 ± 8.28	0.001
	Intervention	20.33 ± 4.40	15.06 ± 6.41	0.006	−5.26 ± 8.34	
NO	Control	9.12 ± 5.88	14.25 ± 9.41	0.060	5.12 ± 11.97	0.525
	Intervention	12.25 ± 7.66	15.00 ± 12.10	0.312	2.75 ± 11.46	
hs-CRP	Control	10508.52 ± 5479.98	9104.60 ± 4901.67	0.142	−1403.91 ± 4425.17	0.785
	Intervention	8953.30 ± 5588.06	7249.86 ± 5091.62	0.007	−1703.43 ± 2770.61	
TNF-*α*	Control	15.95 (15.17, 16.66)	15.87 (14.75, 16.22)	0.330	−11.00 (−2.30, 0.59)	0.276
	Intervention	15.96 (15.16, 16.66)	15.94 (15.44, 16.53)	0.615	0.37 (−1.31, 1.28)	
IL-6	Control	58.24 (55.19, 61.12)	56.57 (53.05, 62.63)	0.738	−2.66 (−7.21, 12.42)	0.196
	Intervention	60.10 (55.99, 73.10)	55.21 (53.39, 60.48)	0.050	−7.27 (−21.33, 1.96)	
Vit E	Control	740.00 ± 104.12	594.07 ± 86.91	0.001	−145.92 ± 157.84	0.218
	Intervention	703.05 ± 73.91	607.86 ± 69.22	0.001	−95.18 ± 102.56	

Values are mean ± SD for the data with normal distribution and median (interquartile ranges) for the data not normally distributed. PCO: protein carbonyl; NO metabolite: nitric oxide metabolite; hs-CRP: high-sensitivity C-reactive protein; IL-6: interleukin-6; TNF-*α*: tumor necrosis factor-*α*; Vit E: vitamin E.

## Data Availability

The data supporting the findings of this study are available within the article.
